# Health Notes in Bristol, 1905

**Published:** 1906-06

**Authors:** D. S. Davies

**Affiliations:** Medical Officer of Health


### HEALTH NOTES IN BRISTOL, 1905.

BY

D. S. Davies, M.D. Lond.,

*Medical Officer of Health.*

The recent publication of the Registrar-General's Annual Summary for 1905 enables some comparison to be made between the mortality figures for Bristol and other Districts in England and Wales.

The *Birth rate* in Bristol for the year 1905, 26.9, shows a slight increase on recent local rates, but compares with a rate of 27.2 for England and Wales, and of 28.2 for the 76 great towns.

The *Death rate* in Bristol for the same period is 14.7, a satisfactorily low rate, and compares with a rate of 15.2 for England and Wales, and of 15.7 for the 76 great towns.

The *Infant Mortality* shows a rate per 1000 births of 122.4, which compares with a rate of 128 for England and Wales, and of 140 for the 76 great towns.

The rate from the *Seven Chief Epidemic Diseases* (Zymotic Rate) was in Bristol 1.6 per 1000 living, and in the 76 great towns was 1.8.

### PREVALENCE OF COMMUNICABLE DISEASE IN BRISTOL IN 1905.

*Scarlet Fever*, which has been much in evidence since 1900, continued to show a satisfactory decrease, the notifications having fallen from 798 per 100,000 in 1902 to 302 in 1905; the case-mortality, 3.5, is somewhat higher than in previous years: the total number of deaths in the whole city was only 39, a striking contrast with the mortality observed in old-time epidemic periods, as for example in 1869-70, when over 900 deaths were recorded in a city of less than half the present size.

*Diphtheria*, which showed a remarkable increase in fatal prevalence from 1900 to 1902, when the deaths corresponded to 54 per 100,000 population, fell to the satisfactory figure of 15 for the year 1905. The decrease in fatality has been accompanied by disappearance of the tendency to form school-outbreaks; probably the major part of the susceptible youthful population has become immunised by the prevalence of the exceedingly mild, often unrecognised, varieties, which form so distinguishing a feature accompanying the prevalence of virulent epidemic diphtheria.

*Enteric Fever*. The returns in regard to this disease are eminently satisfactory. Both notifications and deaths show the lowest figures recorded in the city; the deaths number exactly half those returned in 1904, and correspond to the remarkably low rate of 3 per 100,000 living. The case-mortality was 17 per cent., reckoned on the 76 notified cases.

*Diarrhœa—Infantile Diarrhœa*. The number of deaths returned in 1905 from this disease was 169, compared with 206 in 1904, and with 348 in 1900. The fatality from this cause is largely influenced by the climatic conditions of the late summer and autumn. The diarrhœa rate for Bristol was 47 per 100,000, compared with a rate for the 76 great towns of 83.

*Typhus Fever* continues habitually absent from the city—the last group of cases was in 1889-90. It is true that one case was notified as typhus fever during 1905, but the diagnosis was not confirmed.

*Measles*. This disease, although popularly disregarded, is a periodically recurring cause of regrettable mortality. In 1905 180 deaths were returned from this disease, of which 168 occurred in children under 5. The highest mortality in recent years was in 1902, when 411 deaths were due to measles, the prevalence and fatality of which are intimately bound up with school attendance, and especially with infant classes. In the spring of 1905 the Bristol Education Committee adopted regulations determining the exclusion of scholars from affected houses and the prompt notification of cases from the teachers to the Health Department. Many of the teachers loyally fulfil this duty, but not all, so that the full value of the system is not yet realised. It is in regard to this disease that the value of an organised system of medical school-inspection should be particularly felt; and, if the school-teachers co-operate, lists of those children who have previously suffered from measles may be kept, and exclusion may be safely relaxed in their case, leading to less interference in schoolwork with a reasonable measure of success in prevention of outbreaks, provided notice of initial cases is forwarded at the very earliest opportunity. This has been found to be the case in the experience of the London Education Committee.

*Whooping Cough* also led to considerable mortality, and 123 deaths were ascribed to this cause, of which 121 were in children under 5 years of age.

*Influenza* was credited with 54 deaths in 1905; this cause of death had a very real meaning in the year 1890, when an epidemic wave, travelling westward from Russia, reached England, and caused, directly and indirectly, excessive mortality. The term has continued to appear in the death returns with greater or less regularity ever since, but I am unable to define exactly what it connotes in Bristol at the present time.

*Phthisis.* Notification of this disease commenced in Bristol on September 5th, 1905, and up to the end of the year 330 cases were notified—196 males and 134 females. The Winsley Sanatorium was opened in December, 1904, and the first Bristol case was admitted on February 27th, 1905. Sixty-four cases had been admitted to the Bristol maintained beds up to the end of the year 1905—38 males and 26 females. In very many of these cases considerable improvement was reported; and the educational influence of the Institution is beyond praise, and should effectually add to the safeguarding of the public health by spreading true ideas as to preventive home treatment.

*Smallpox.* This disease was prevalent in one or two neighbouring districts, and several "sample" cases were introduced. The extreme mildness of the variety led to some difficulty in tracing cases, and at one time, in June, it appeared that a widespread outbreak was inevitable, as a man with the eruption out on him had sat drinking for some hours with many others. Special vigilance detected one or two contact cases, which proved to be all in this district; whether the others, if any, developed in neighbouring districts, or whether the slight infectivity of this variety failed to continue the disease, cannot be certainly stated.

